# MicroRNA-710 regulates multiple pathways of carcinogenesis in murine metastatic breast cancer

**DOI:** 10.1371/journal.pone.0226356

**Published:** 2019-12-13

**Authors:** Byunghee Yoo, Nikhil Meka, Patrick Sheedy, Ann-Marie Billig, Pamela Pantazopoulos, Zdravka Medarova

**Affiliations:** 1 MGH/MIT/HMS Athinoula A. Martinos Center for Biomedical Imaging, Massachusetts General Hospital and Harvard Medical School, Boston, MA, United States of America; 2 College of Arts and Science, New York University, NY, United States of America; 3 Department of Health Sciences, CaNCURE Program, Northeastern University, Boston, MA; University of Pécs Medical School, HUNGARY

## Abstract

Prior research has shown that critical differences between non-metastatic and metastatic tumor cells are at the level of microRNA. Consequently, harnessing these molecules for the treatment of metastatic cancer could have significant clinical impact. In the present study, we set out to identify metastasis-specific microRNAs which drive metastatic colonization of distant organs. Using a murine model of metastatic breast cancer, we employed a directed approach in which we screened for microRNAs that are differentially expressed between the primary tumors and metastatic lesions but concordantly expressed in all of the metastatic lesions irrespective of the tissue that is colonized. Of the identified targets, we focused on miR-710, which was consistently and significantly downregulated in the metastatic lesions relative to the primary tumors. The level of downregulation was independent of the distant organ that is involved, suggesting that miR-710 plays a fundamental role in metastatic colonization. Computational target prediction suggested a pleiotropic role for miR-710 in apoptosis, migration and invasion, and stemness. Using a previously validated oligonucleotide delivery system, we introduced miR-710 mimics into 4T1 metastatic breast adenocarcinoma cells and assessed the resultant phenotypic effects. We demonstrated significant inhibition of cell viability, migration, and invasion. We also showed that the treatment profoundly enhanced cell senescence, reduced stemness, and influenced markers of epithelial to mesenchymal transition, as evidenced by enhanced E-cadherin and reduced vimentin expression. This knowledge represents a first step towards harnessing a similar approach to discover novel microRNA targets with therapeutic potential in metastasis.

## Introduction

The recent past has seen impressive progress in the field of cancer therapy. Still, the outcomes for people diagnosed with advanced metastatic cancer are poor. These poor outcomes highlight the need to develop strategies different from the traditional cytotoxic approach to cancer therapy that has dominated the field over the past century.

One key target class of molecules that hold yet unfulfilled promise are microRNAs. Prior research has shown that some critical differences between non-metastatic and metastatic cells are at the level of microRNA [1]. Indeed, microRNAs have emerged as valuable biomarkers of malignancy. Consequently, harnessing these molecules for the treatment of metastatic cancer could have a significant clinical impact.

Given the robustness of microRNAs as determinants of tumor-cell phenotype, their high level of “druggability”, and the potential for modular rational therapeutic design based on the phenomenon of complementarity, there has been a lot of interest in harnessing microRNAs for cancer therapy.

Our own research to date has helped establish miRNA-10b as a pro-metastatic microRNA target [2] which drives the context-dependent survival and proliferation of tumor cells as a function of their microenvironment [3]. Based on this knowledge, we have designed modular nanodrugs that can reprogram these microRNAs following intravenous injection, resulting in durable regressions of established metastases [3–7].

The goal of the present study was to identify and validate novel therapeutic targets against metastases based on the knowledge that the metastatic tumor cell relies on unique adaptive mechanisms to survive outside of the primary tumor microenvironment and to colonize a distant organ. Using an immunocompetent murine model of metastatic breast cancer, we have used a directed approach to discover novel microRNA targets that play fundamental roles in metastatic colonization and are not organ-specific. Unlike traditional approaches that look for highly expressed microRNAs in metastatic cells or tissues, our strategy was to focus on microRNAs that are differentially expressed between the primary tumors and metastatic lesions in a given model and that show a high level of concordance between different metastatic organs. This approach would allow us to discover important microRNA targets that do not undergo profound changes of expression in the course of metastasis and metastatic colonization but are nevertheless critical and fundamental drivers of the metastatic process.

Using this rationale, we identified miRNA-710 as an important target in metastatic colonization that can be targeted for therapy using modular nanodrugs. We showed that miR-710 is downregulated in the metastatic lesions relative to the primary tumors in animal models of breast cancer, suggesting a role in metastatic colonization. We demonstrated that miR-710 expression is independent of the organ site of metastasis, suggesting a fundamental role. miR-710 computational target prediction suggested a pleiotropic role in apoptosis, migration, invasion, senescence, and stemness in murine as well as human cells. Finally, we showed that miR-710 enrichment in metastatic cells using a MN-miRNA nanodrug profoundly affects multiple aspects of the metastatic cell phenotype, suggesting that similar approaches for the upregulation of miR-710 in metastatic lesions can be designed to have robust therapeutic effects *in vivo*. Given that miR-710 is not an annotated human microRNA but is predicted to affect the expression of multiple human genes related to carcinogenesis, our results imply a strategy for cancer therapy based on poorly conserved microRNAs with no human homologues.

## Methods

### Animal model

Four female Balb/c mice (8 weeks old, The Jackson Laboratory; Bar Harbor, ME) were implanted orthotopically under the top right third mammary fat pad with the 4T1-Red-Fluc cell line (0.5 x 10^6^ cells). The cells express luciferase and can be detected by noninvasive bioluminescence imaging (BLI) for analysis of tumor and metastatic burden. All animals were scanned weekly by BLI in order to monitor tumor growth and metastasis. Distant metastases and primary tumors were harvested using BLI-guided macrodissection.

Animals were sedated under Isoflurane anesthesia 10 minutes before cell implantation, i.v. injection, or imaging. For post-procedural pain relief, animals were administered buprenorphine twice daily for the duration of the experiment (i.e., approximately two weeks post-inoculation). All procedures were carried out in aseptic fashion in clean facilities. Animals were observed daily for their level of activity and normal eating, drinking and grooming behavior. If an animal demonstrated marked weight loss, and/or showed difficulty walking, it was removed from the study and sacrificed according to AVMA guidelines using a high dose of sodium pentobarbital injected intraperitoneally (200 mg/kg IP). Animals were housed in the MGH Animal Facility, which is under the supervision of the MGH Office of Laboratory Animal Research. All animal experiments were performed in compliance with institutional guidelines and approved by the Institutional Animal Care and Use Committee at Massachusetts General Hospital (Boston, MA).

### Bioluminescence optical imaging (BLI)

BLI was used to evaluate metastatic burden. Anesthetized mice were injected in the lower left abdominal quadrant with D-luciferin potassium salt in DPBS (200 μL of 15 mg/mL; Perkin Elmer, Hopkinton, MA) 12 minutes before image acquisition. Identical imaging acquisition settings (time, ~ 0.5–60 seconds; F-stop, 2; binning, medium) and the same region of interest (ROI) were used to obtain total radiance (photons/sec/cm^2^/sr). BLI (IVIS Spectrum, Perkin Elmer, Hopkinton, MA) was performed for about 6 to 12 minutes to obtain the maximum radiance from the lesions. The primary tumors were masked to avoid BLI signal saturation. All images were processed using Living Image Software (ver 4.5, IVIS Spectrum, Perkin Elmer, Hopkinton, MA).

### Microarray analysis

For the microarray analysis, primary tumors and distant metastases were processed to obtain a miRNA enriched solution using a miRNeasy mini kit, according to the manufacturer’s protocol (Qiagen Inc., Hilden, Germany). Differential miR expression analysis was performed by Exiqon using their miRCURY LNA^™^ microRNA Array. All experiments were conducted at Exiqon Services, Denmark. The quality of the total RNA was verified by an Agilent 2100 Bioanalyzer profile. 750 ng of total RNA from both sample and reference was labeled with Hy3^™^ and Hy5^™^, respectively, using the miRCURY LNA^™^ microRNA Hi-Power Labeling Kit, Hy3^™^/Hy5^™^ (Exiqon, Denmark), following the procedure described by the manufacturer. The Hy3^™^-labeled samples and a Hy5^™^-labeled reference RNA sample were mixed pair-wise and hybridized to the miRCURY LNA^™^ microRNA Array 7th Gen (Exiqon, Denmark), which contains capture probes targeting all murine microRNAs in the miRBASE 18.0. The hybridization was performed according to the miRCURY LNA^™^ microRNA Array Instruction manual using a Tecan HS4800^™^ hybridization station (Tecan, Austria). The miRCURY LNA^™^ microRNA Array slides were scanned using the Agilent G2565BA Microarray Scanner System (Agilent Technologies, Inc., USA) and the image analysis was carried out using the Nexus Expression (Nexus Copy Number Discovery Ver.10.0. BioDiscovery Inc., El Segundo, CA).

### Bioinformatics analysis

Gene targets of selected microRNAs were predicted using TargetScan [8] and MiRDB [9, 10]. Only targets with cumulative weighted context scores < -0.2 were used for analysis. Functional annotation of the predicted targets was carried out using the Database for Annotation, Visualization and Integrated Discovery (DAVID) v6.8 [11, 12]. The Kyoto Encyclopedia of Genes and Genomes (KEGG) Pathways in Cancer map was used to demarcate target genes relevant to cancer.

### Preparation of MN-miRNA

The sequence of the miRNA-710 mimic (miR710; MW = 14,018.5 g/mol) consisted of 5’-S-S-GGUUCAGAACCCCUCUCAACUC (passenger sequence) and 5’- CCAAGUCUUGGGGAGAGUUGAG (miR710, guide sequence). The sequence of the scrambled miRNA oligo (SCRmiR; MW = 14,647.3 g/mol) was 5’-S-S-AGUGUUGGAGGAUCUUUCUCAUCU (passenger sequence) and 5’-UCACAACCUCCUAGAAAGAGUAGA (SCRmiR, guide sequence). Both miRNAs were designed and synthesized by Dharmacon (Laffayette, CO). The 5’-disulfide modification was introduced into the passenger strands for conjugation to magnetic nanoparticles (MN).

Following previously described protocols [4, 6], MN was conjugated to the heterobifunctional linker N-Succinimidyl 3-[2-pyridyldithio]-propionate (SPDP, Thermoscientific Co., Rockford, IL), which was utilized for the linkage of activated miRNA oligos. SPDP was dissolved in anhydrous DMSO and incubated with MN, which has a pyridyldithio group to form a reduction labile disulfide linkage with the miRNA oligos. The 5’-disulfide capping of the siRNA oligo was activated to release the thiol via 3% TCEP (Thermoscientific Co., Rockford, IL) treatment in nuclease free PBS. The miRNA oligos were purified by an ammonium acetate/ethanol precipitation method. After TCEP-activation and purification, each oligo (miR710 and SCRmiR) was dissolved in nuclease-free water and incubated with the SPDP modified MN overnight to prepare nanodrugs (MN-miR710 and MN-SCRmiR). Nanodrugs were freshly prepared each week and characterized to determine the number of miRNAs per MN, the hydrodynamic size of the nanodrugs as well as their Zeta-potential (Zetasizer Nano ZS, Malvern, UK).

### Cell culture

The luciferase-expressing 4T1-Red-Fluc breast cancer cell line, derived originally from mouse mammary gland adenocarcinoma, was obtained from Perkin Elmer (Hopkinton, MA). The tumor growth and metastasis of 4T1 cells in BALB/c mice reflect those in humans and are widely used as a model for stage IV human breast cancer. The cells were cultured in high-glucose DMEM supplemented with 5% FBS and antibiotics (100 units/mL penicillin and 100 mg/mL streptomycin), as recommended by the supplier.

### Migration assay

4T1 cells were serum-starved for 1 day. Following, a cell suspension containing 1.0 x 10^6^ cells/mL was prepared in serum free media. 500 μL of media containing 10% fetal bovine serum was added to the bottom well of the migration plate. 200 μL of the cell suspension was added to the inside of each insert. MN-miR710, MN-SCRmiR, or MN were added in the inserts and the final volume was adjusted to 300 μL for a final oligo concentration of either 1 or 2 μM. The cell suspension plates were incubated for 24 hours and the inserts were transferred to clean wells containing 225 μL of Cell Detachment Solution. This was followed by 30 minutes of incubation at 37º. Finally, 75 μL of 4X Lysis Buffer/CyQuant^®^ GR Dye solution (a 1:75 dye dilution in 4X Lysis Buffer) was added to each well and the cells were incubated for 20 minutes at room temperature. 200 μL of the mixture was transferred to a 96-well plate appropriate for fluorescence measurement. Fluorescence intensity was quantified using a fluorescence plate reader at 480 nm/520 nm.

### Invasion assay

The invasion chamber plate was equilibrated to room temperature for 10 minutes under sterile conditions. The basement membrane layer of the cell culture inserts was rehydrated by adding 200 μL of warm, serum-free media to the inner compartment. The rehydration medium was removed from the inserts after 1 hr. 500 μL of media containing 10% fetal bovine serum was added to the bottom well of the migration plate. 200 μL of cell suspension solution (1.0 x 10^6^ cells/mL) was added to the inside of each insert. MN-miR710, MN-SCRmiR, or MN were added to the inserts and the final volume was adjusted to 300 μL for a final oligo concentration of 1 or 2 μM. After 48 hours, the inserts were transferred to clean wells containing 225 μL of Cell Detachment Solution. The inserts were incubated for 30 minutes at 37º and the cells were dislodged from the underside of the membrane. Finally, 75 μL of 4X Lysis Buffer/CyQuant^®^ GR Dye solution (a 1:75 dye dilution in 4X Lysis Buffer) was added to each well with cells and 225 μL of Cell Detachment Solution. The cells were incubated for 20 minutes at room temperature. 200 μL of the mixture was transferred to a 96-well plate appropriate for fluorescence measurement. Fluorescence intensity was quantified using a fluorescence plate reader at 480 nm/520 nm.

### Senescence

Cells (1x10^6^) were seeded in a 24-well plate. MN-miR710, MN-SCRmiR, or MN were added in each well for a 1 μM final oligo concentration. The cells were incubated for 48 hours (37º C, 5% CO_2_), trypsinized, washed, and resuspended in 500 μL of tissue culture media. The media was replaced with 500 μL of fresh media containing 1.5 μL of senescence dye (Senescence assay kit, Abcam, Cambridge, MA), followed by incubation for 1–2 hours (37º C, 5% CO_2_). The cells were washed twice and analyzed immediately using flow cytometry. Flow cytometry was performed using BD LSRFortessa X-20 (BD Biosciences, San Jose, CA) with 100,000 cells per acquisition. Levels of senescence were quantified by monitoring the expression level of beta galactosidase.

### Stemness

Cells (1.0 x 10^6^) were seeded in a 24-well plate. MN-miR710, MN-SCRmiR, or MN were added in each well for a 1 μM final oligo concentration. The cells were incubated for 48 hours (37º C, 5% CO_2_), trypsinized, washed, and resuspended in 500 μL of culture media. 5 μL of the activated ALDEFLUOR^TM^ Reagent (Aldefluor kit, STEMCELL Technology, Cambridge, MA) per milliliter of sample was added to 1.0 mL of cell suspension. The suspended cells were incubated for 1 hour at 37º, washed twice, and analyzed immediately using flow cytometry. The degree of cell stemness was quantified by monitoring the expression level of aldehyde dehydrogenase.

### Biomarker analysis

The cells were trypsinized, washed twice, and resuspended in cold FACS buffer (PBS, 0.5–1.0% BSA or 5–10% FBS, 0.1% NaN_3_ sodium azide) to achieve a single cell suspension. 1 x 10^6^ cells/mL were utilized to stain for each biomarker. To minimize non-specific binding and background signal, the cells were treated with mouse serum in FACS buffer on ice for 20 min. After washing, the cells were treated with a primary antibody against each biomarker. The cells were washed 3 times and treated with corresponding secondary antibody for 1 hr and analyzed immediately using flow cytometry. For the analysis of vimentin and E-cadherin, primary antibodies (ab92547 and ab11512, ABCam, Cambridge, MA) were utilized and secondary antibodies (ab96899 and ab98420) were selected in accordance with the species of origin of the primary antibodies.

### Cell viability assay

Cells (5x10^3^) were seeded in a 96-well plate (n = 3) and incubated for 24 hrs. MN-miR710, MN-SCRmiR, or MN were added in each well for a final oligo concentration of 0.01, 0.1, 0.5, 1.0, 2.5, 5.0 and 10 μM. This treatment was used to determine the IC50 of MN-miR710. In addition, the same concentrations of nanodrug were added together with Paclitaxel at 0.01, 0.1, 0.5, 1.0, 2.0, 5.0, 10, 20, and 50 μM. After 48 hrs of incubation, the cells were washed with HBSS. 90 μL of the culture media and 10 μL of MTT solution (3-(4,5-dimethylthiazol-2,5-diphenyltetrazolium bromide, 5mg/mL) were added to each well. After 4 hrs of incubation, the cells were washed with DPBS twice, and suspended in DMSO. Fluorescence intensity was measured at 570 nm with a reference of 630 nm to analyze cell survival.

### Statistical analysis

Data were expressed as mean ± s.d.. Statistical comparisons were made using a two-tailed t-test (SigmaStat 3.0; Systat Software, Richmond, CA). A value of P < 0.05 was considered statistically significant.

## Results

### miR-710 is computationally predicted to play a fundamental role in metastasis

**Fig 1:**
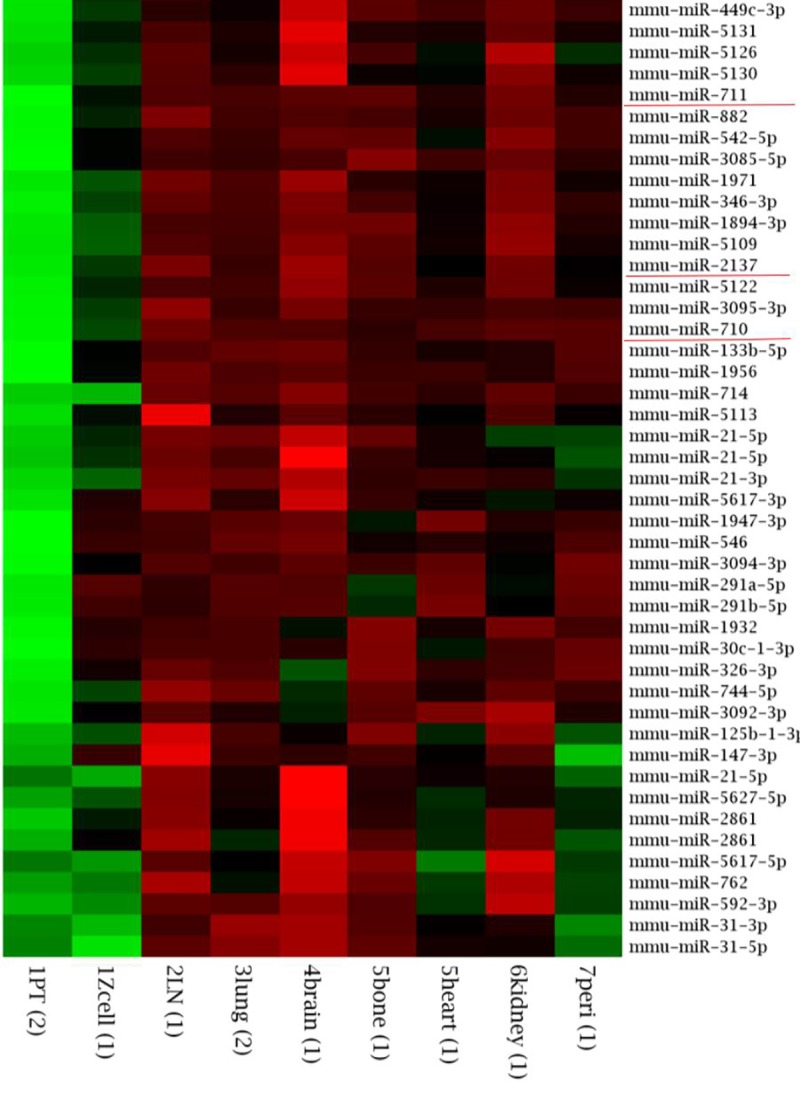
Heatmap of the 45 most highly differentially expressed miRNAs in the metastatic lesions relative to the primary tumors of mice bearing metastatic breast cancer isografts. Metastatic lesions were macro-dissected from the lymph nodes (LN), lungs, brain, bone, heart, kidney, and peritoneum.

In order to identify microRNA targets that play fundamental and organ-independent roles in metastatic colonization, we screened for miRNAs whose expression in the metastatic lesions was different from that in the primary tumor samples but concordant among the different metastatic lesions, which included lung, lymph node, bone, brain, heart, kidney, and peritoneum. We identified surprisingly few targets that were significantly differentially expressed between the primary and metastatic tissues but not differentially expressed between different metastatic tissues. Specifically, we found 4 highly differentially expressed microRNAs (log > 2) that were predicted to play a role in apoptosis (miR-711), differentiation (miR-2137), general tumor suppression (miR-542-5p), and apoptosis, invasion, and stem cell renewal (miR-710) (Fig 1, S1 Fig). Whereas a number of microRNA targets were expressed at log >2, they did not meet selection criteria because that level of expression was not concordant to the same degree of significance in all of the metastatic lesions. miR-542-5p was excluded because it did not constitute a novel target.

**Fig 2:**
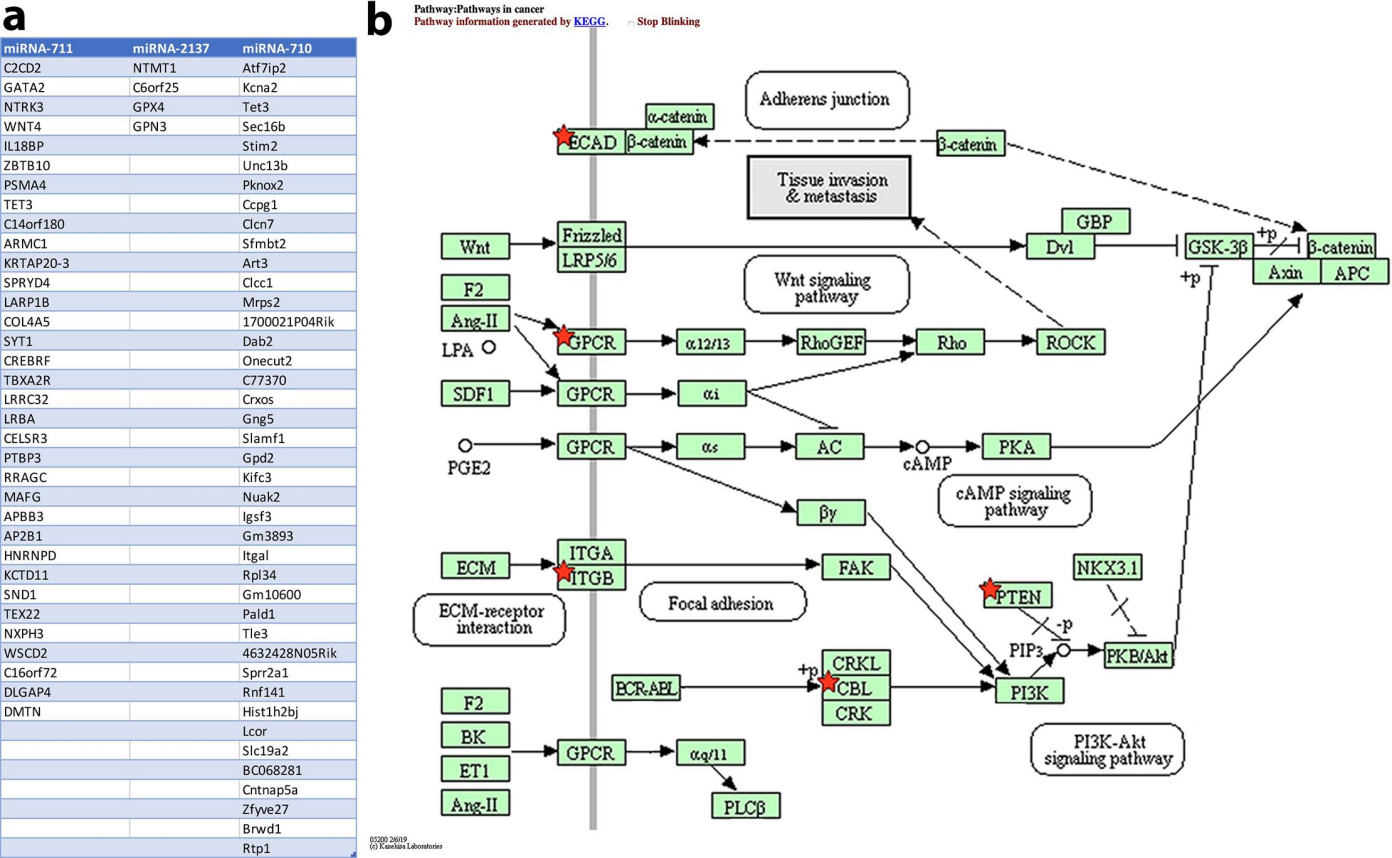
List of predicted top murine targets of mmu-miR-711, mmu-miR-2137, and mmu-miR-710. **a.** The predicted targets of miR-711 clustered with genes related to apoptosis, angiogenesis, cell adhesion, and immune regulation. The predicted targets of miRNA-2137 clustered with drug resistance and proliferation. The predicted targets of miR-710 clustered with apoptosis, cell adhesion and motility, cytoskeletal structure, and stemness. **b.** Functional annotation clustering of mmu-miR-710 predicted murine targets centered around tissue invasion and metastasis. The KEGG Pathways in Cancer annotation cluster is shown with the predicted targets marked by red stars.

The TargetScan online tool (Whitehead Institute for Biomedical Research) was used to predict targets of each of the remaining three candidate microRNAs. The predicted targets of miR-711 clustered with genes related to apoptosis, angiogenesis, cell adhesion, and immune regulation. The predicted targets of miRNA-2137 clustered with drug resistance and proliferation. The predicted targets of miR-710 clustered with apoptosis, cell adhesion and motility, cytoskeletal structure, and stemness (Fig 2, S2 File and S2 Fig).

Based on these results, we focused on miR-710 as the target in our subsequent studies. Our choice was based on the high level of differential expression of miR-710 between the primary tumors and matched metastatic lesions, the high degree of concordance in the expression of miR-710 among metastatic lesions from different organ sites, the fact that miR-710 represents a novel target in metastasis, and the predicted large pleiotropic effects of miR-710 on multiple aspects of carcinogenesis and metastasis.

**Fig 3:**
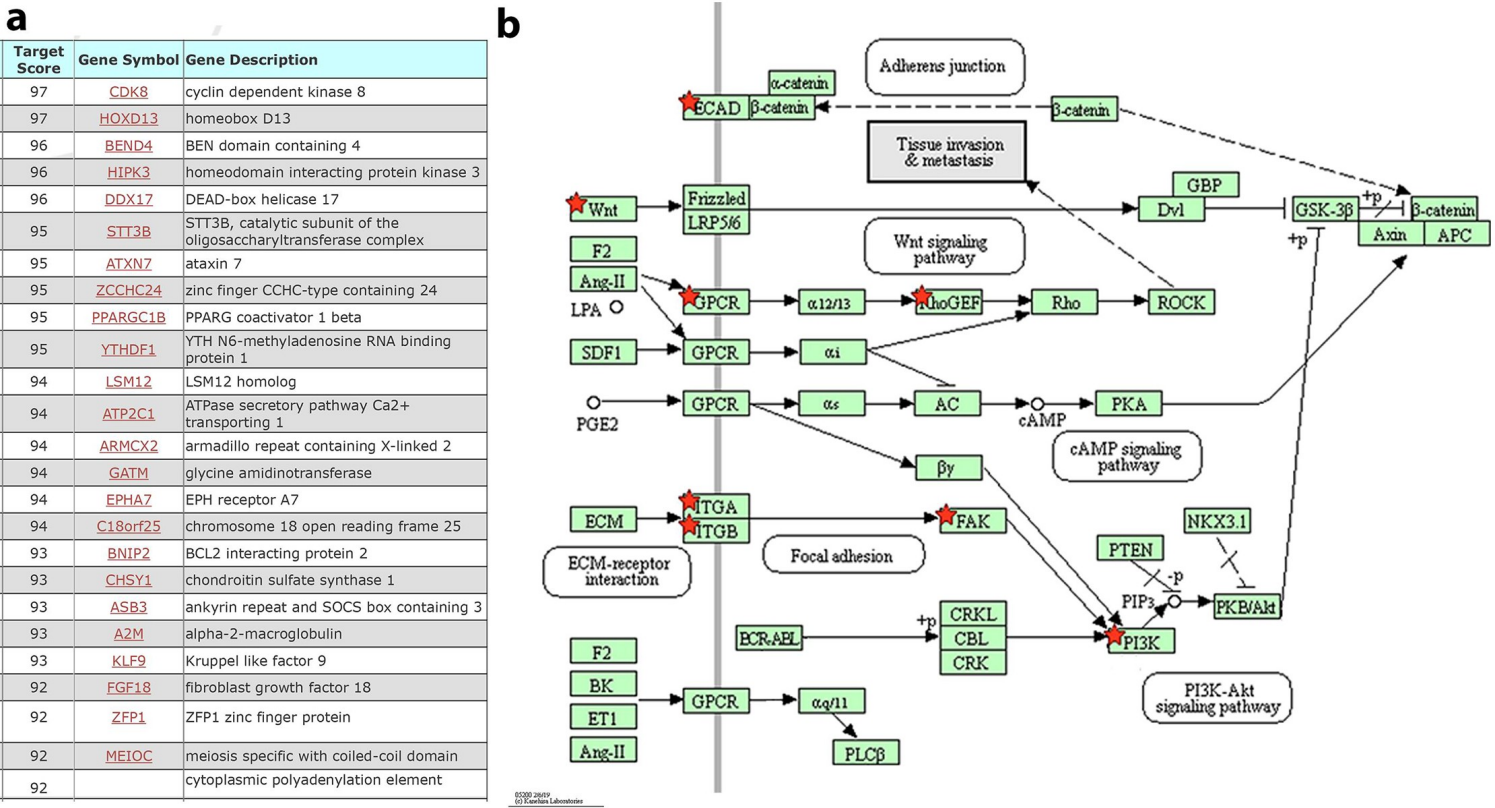
List of predicted top human targets of mmu-miR-710. **a.** We queried the human miRDB database against the mature mmu-miR-710 sequence and found 508 predicted targets. related to cell cycle, cytoskeletal remodeling, invasion and migration, and stemness. **b.** Functional annotation clustering of miR-710 predicted human targets centered around tissue invasion and metastasis. The KEGG Pathways in Cancer annotation cluster is shown with the predicted targets marked by red stars.

Given that the candidate miRNA targets were identified in a murine cell line and that miR-710 is not an annotated human miR, we also validated the role for a putative human miR-710 ortholog using bioinformatics. We queried the miRDB database against the mature mmu-miR-710 sequence and found 508 predicted targets related to cell cycle, cytoskeletal remodeling, invasion and migration, and stemness (Fig 3, S2 File and S3 Fig).

### Synthesis of MN-miR710 and MN-SCRmiR

**Fig 4:**
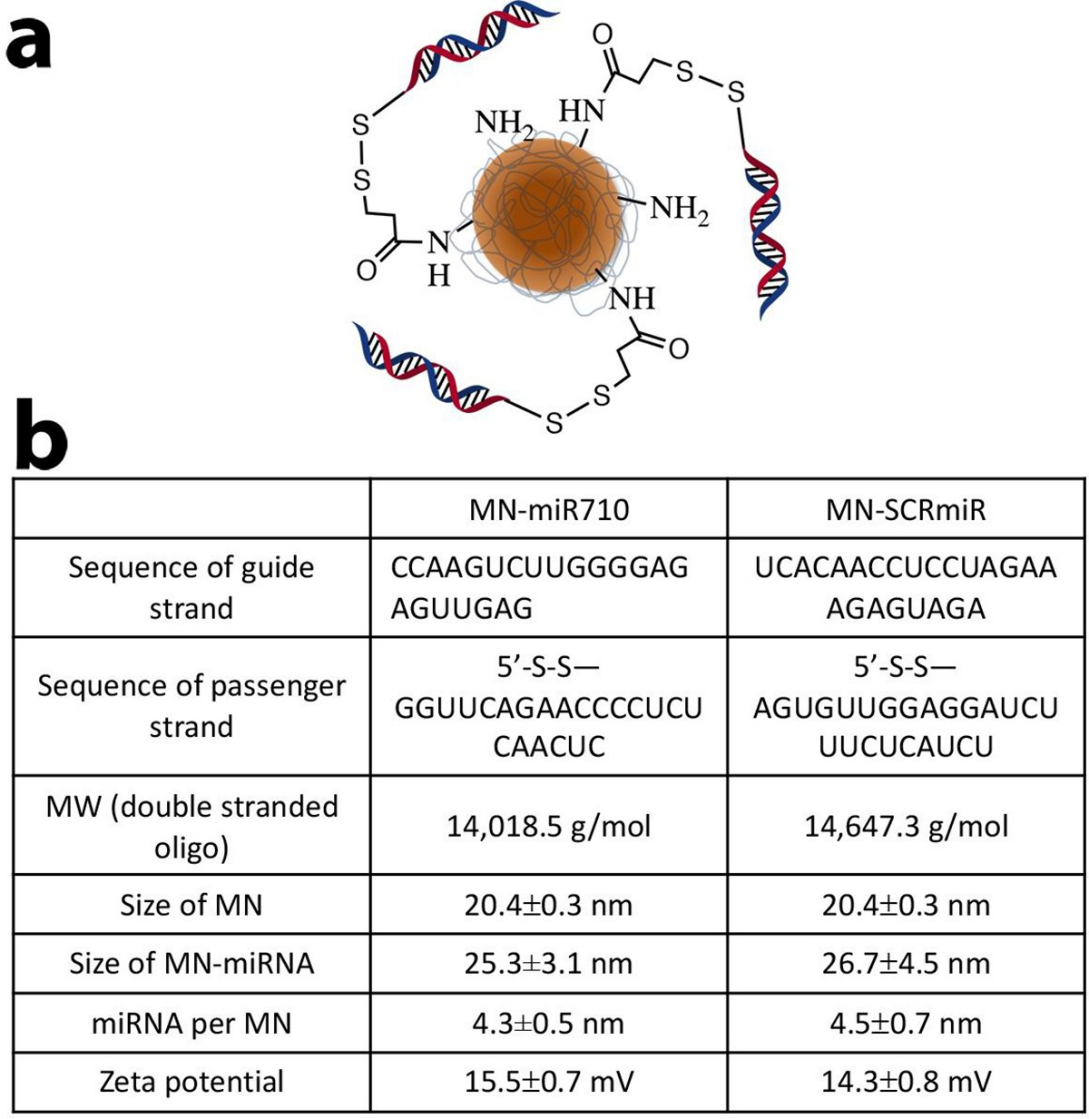
Structure and characterization of MN-miR710. **a.** The nanodrug consists of iron oxide nanoparticles (MN) conjugated to miR-710 mimics. **b.** Characterization of MN-miR710 and the scrambled control, MN-SCRmiR.

To determine the phenotypic role of miR-710, we constructed an MN-miR710 nanodrug by conjugating a miR-710 oligonucleotide mimic to dextran-coated iron oxide nanoparticles (MN) (Fig 4A), as previously described [4, 13]. The number of miRNA oligos per MN was maintained at 4.3±0.5 for MN-miR710 and 4.5±0.7 for MN-SCRmiR, which was quantified by the electrophoresis analysis method [13]. The size of the nanodrugs was determined as 25.3±3.1 nm for MN-miR710 and 26.7±4.5 nm for MN-SCRmiR. The Zeta-potential of the nanodrugs was measured as 15.5±0.7 mV for MN-miR710 and 14.3±0.8 mV for MN-SCRmiR (Fig 4B).

### Phenotypic effects of MN-miR710

**Fig 5:**
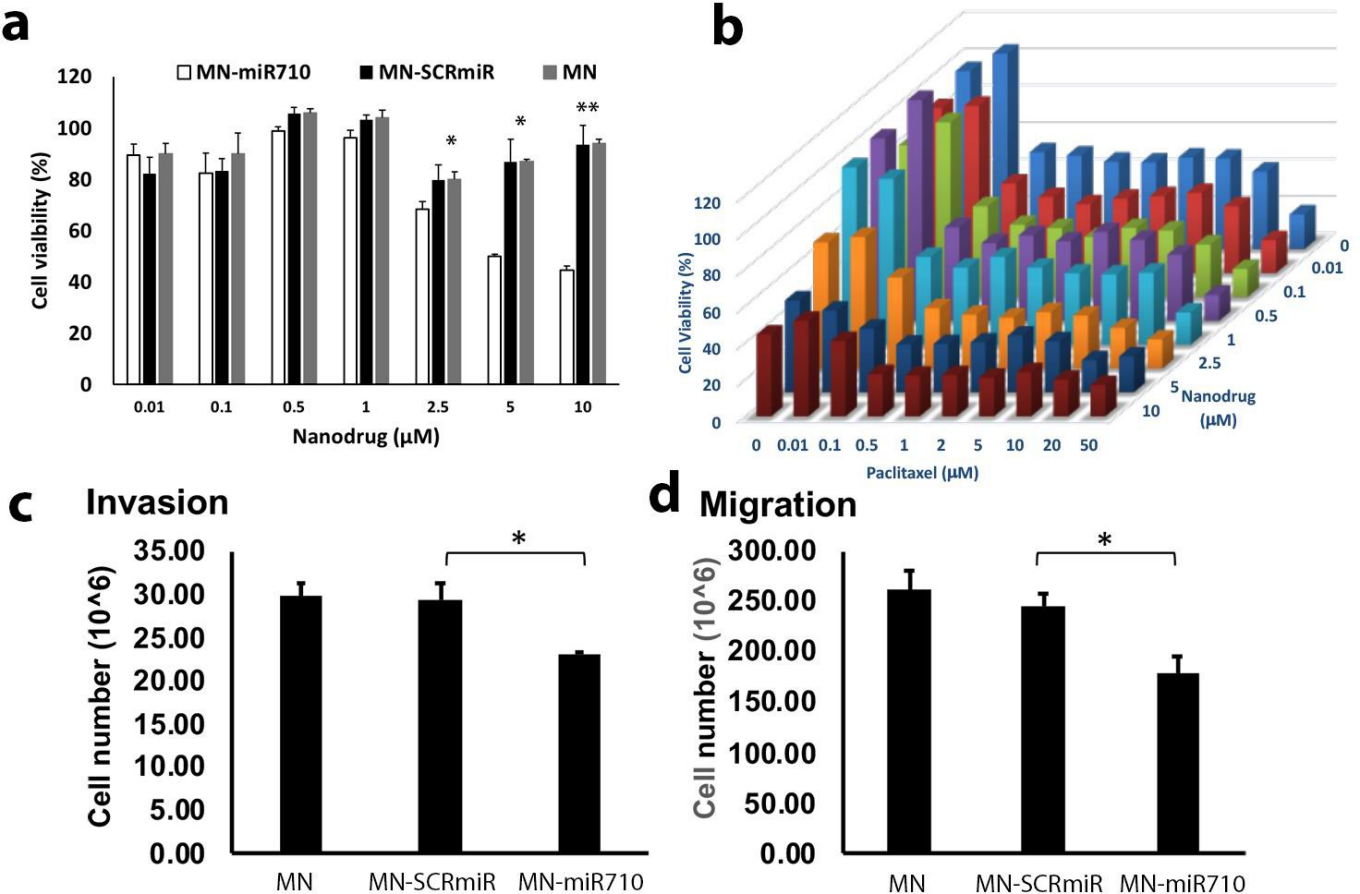
The effect of MN-miR710 on the viability, migration, and invasion of metastatic breast adenocarcinoma cells. **a.** MN-miR710, demonstrated an IC50 of 5 μM. **b.** In combination with paclitaxel, MN-miR710 potentiated the effect of paclitaxel on viability and reduced the IC50 for paclitaxel from 4.77 μM to 0.6 μM. **c.** MN-miR710 had a significant effect on the invasion of 4T1 cells, as compared to the MN-SCRmiR control. **d.** MN-miR710 had a significant effect on the migration of 4T1 cells, as compared to the MN-SCRmiR control. Student’s t-test, n = 3; *, p < 0.05; **, p < 0.01.

We incubated murine 4t1 metastatic breast adenocarcinoma cells with increasing concentrations of MN-miR710 alone or in combination with paclitaxel and analyzed cell viability (Fig 5). When MN-miR710 was administered alone, cell viability was not significantly different from the control treatments at concentrations below 5.0 μM (expressed as oligonucleotide) (Fig 5A). When MN-miR710 was co-administered with paclitaxel, cell viability was decreased dramatically in comparison to paclitaxel alone, indicating that MN-miR-710 enhanced the chemosensitivity of the cell line to the chemotherapeutic. Specifically, the IC50 of Paclitaxel alone was determined as 4.7 μM. It was reduced to 0.6 μM in combination with 1 μM MN-miR710 (Fig 5B). As expected, similarly to MN-SCRmiR, the MN nanoparticles by themselves did not influence cell viability, indicating that the effect was specific to the miR-710 target.

Next, we investigated the effect of MN-miR-710 on the invasion and migration of 4T1 cells. The cells were incubated with MN only, MN-miR710, or MN-SCRmiR for 24 hrs. The invasive capability of 4T1 cells was inhibited by 26% after the treatment with MN-miR710 at an oligo concentration of 2 μM (Fig 5C). Migration was also suppressed by 30% at the same concentration (Fig 5D). No significant effects were observed at a 1 μM oligo concentration.

**Fig 6:**
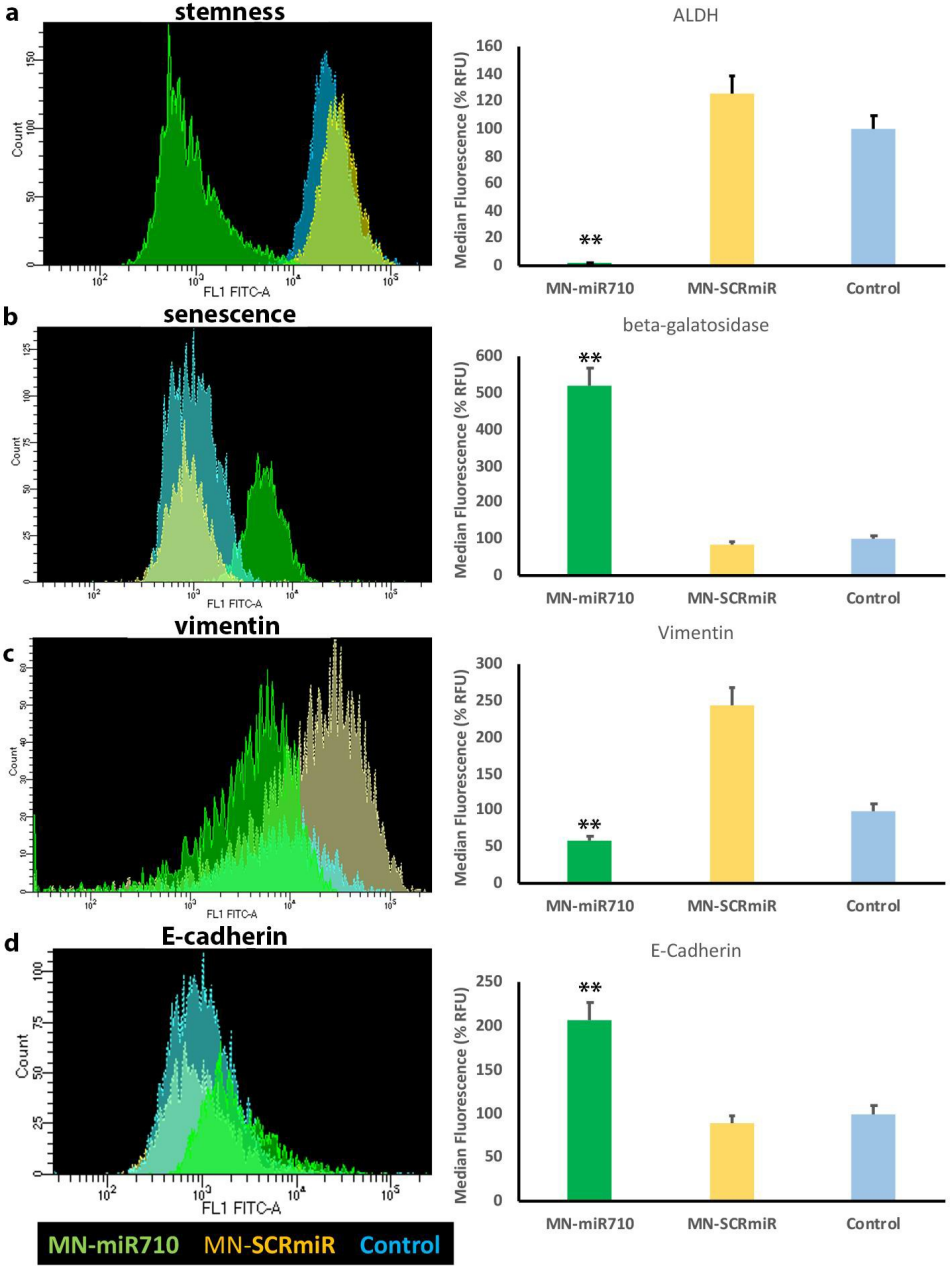
The effect of MN-miR710 on stemness, senescence, vimentin, and E-cadherin expression in metastatic breast adenocarcinoma cells. **a.** MN-miR710 reduced the expression of aldehyde dehydrogenase, as compared to the MN-SCRmiR control, indicating inhibition of stemness. **b.** MN-miR710 treatment led to upregulation of beta-galactosidase relative to the MN-SCRmiR control, reflecting induction of senescence. **c.** Vimentin (mesenchymal biomarker) was down-regulated by MN-miR710 treatment relative to the MN-SCRmiR control. **d.** The levels of E-cadherin (epithelial biomarker) were increased by treatment with MN-miR710 relative to the MN-SCRmiR control. Student’s t-test, n = 3; *, p < 0.05; **, p < 0.01. Data were expressed as % RFU of unstained control.

Finally, we looked at biomarkers of stemness, senescence, and epithelial-to-mesenchymal transition (EMT). After treatment with 1 μM concentration of MN-miR710, the expression of aldehyde dehydrogenase (ALDH), as a marker of stemness was remarkably down-regulated (Fig 6A). In addition, MN-miR710 treatment caused the up-regulation of beta-galactosidase in metastatic breast cancer cells, indicating induction of senescence (Fig 6B). Finally, the expression of E-cadherin and vimentin, as markers of epithelial to mesenchymal transition (EMT), was affected by treatment with MN-miR710. Whereas vimentin was significantly down-regulated (Fig 6C), E-cadherin was induced (Fig 6D). The observed strong effects of MN-miR710 on multiple phenotypic features of the cells supported the potential of miR-710 as a therapeutic target in metastasis.

A noteworthy observation is the difference in the doses of MN-miR710 required to induce a significant phenotypic effect on stemness, senescence, and EMT vs. motility. These differences suggest a differential threshold at which miR-710 influences downstream targets related to stemness vs. migration and invasion and, conceivably, could reflect differences in the stoichiometry between the miRNA effector and downstream mRNA targets relevant to each phenotype.

## Discussion

The present study uses a combination of animal studies, bioinformatics analysis, and in vitro testing in order to identify new microRNA targets in metastasis. We employ a directed approach by screening for microRNAs that are not necessarily highly differentially expressed between the metastatic lesions and the primary tumors but that show concordant differential expression in the metastatic lesions, irrespective of which organ they are derived from. This approach would be biased towards finding microRNAs that play a more fundamental role in metastatic colonization, that are not organ-specific, and that are not necessarily highly abundant.

The rationale for this approach is based on the knowledge that metastases are seeded by rare tumor cells with unique properties, which may function as stem cells in their ability to initiate and propagate metastasis. This selective process causes differences in the epigenetic and ncRNA profiles between primary tumors and metastases. As an example, recent research showed that metastatic tumors are enriched for genes related to proliferation and migration [14]. Also, single cell analysis showed that a stem-like gene expression signature could be uniquely found in metastatic cells [15]. Our own work has found dramatic differences in the expression of select miRNAs in the metastatic lesions vs. primary tumors [6]. Finally, differences in miRNA expression between the primary and metastatic lesions in patients currently represent a key method for the identification of clinical ncRNA biomarkers of metastasis [16].

Our strategy identified mmu-miR-710 as a relevant target in murine metastatic breast cancer. We showed that nanoparticle-based delivery of mmu-miR710 mimics (MN-miR710) to tumor cells, using a previously validated approach [4–7, 13, 17], pleiotropically affected multiple aspects of the cells’ phenotype, including cell viability, migration, invasion, senescence, stemness, and EMT. Combination treatment with paclitaxel indicated that MN-miR710 could synergize with chemotherapy to dramatically potentiate its effects on cell viability. These effects were profound pointing towards the potential of a future miR-710-based therapeutic strategy against metastatic breast cancer.

In trying to evaluate the clinical relevance of our findings, we focused on the fact that mmu-miR-710 does not have an annotated homologue in the human microRNAome and its mature sequence does not appear to be conserved. In addition, miR-710 is unique in its full array of targets within the murine and human transcriptome. For example, mmu-miR-710 has been shown to enhance the differentiation of murine embryonic stem cells [18]. Importantly, studies in growth hormone receptor knock out mice (GHR-KO), discovered that miR-710 is at the core of a three-dimensional lncRNA–mRNA–miRNA regulatory network that defines phenotype in the GHR-KO animals, which is characterized by resistance to cancer and enhanced longevity [19]. Further support for a fundamental role of miR-710 in cancer derives from the fact that its predicted targets include ADAM10, PDGFRA, and FZD7, which are linked to senescence and stemness [20–22].

Given the broad-based and significant effect of miR-710 enrichment in murine metastatic breast cancer cells and the overlap in murine and human targets that are predicted to be influenced by the mature mmu-miR-710, our findings suggest the possibility of employing miRNAs that are not naturally present in the genome for therapy. One can envision a scenario where computationally designed microRNA sequences are selected to inhibit the expression of an array of genes of interest that share unique homologies in their untranslated regions.

Finally, the present work extends a series of studies on the utility of the previously described MN nanoparticle platform for the delivery of RNA interference-based oligonucleotides to tumor cells. While the prior literature has explored the application of these nanoparticles for the delivery of siRNA [17, 23–25] or antagomirs [3–7, 13], here we demonstrate further utility for the delivery of functional miRNA mimics, underscoring the potential of the platform as a therapeutic tool.

This nanoparticle platform could be extended to target microRNAs which play a clear tumor-suppressive role in various aspects of metastasis, including regulation of cancer stem cell properties, epithelial-mesenchymal transition (EMT), microenvironment, and exosome secretion.

Potential targets include miR-206, miR-335, and miR-126 [26], which regulate apoptosis, migration and invasion [27], as well as mesenchymal stem cell and inflammatory monocyte recruitment to the tumor microenvironment [28]. Other targets include miRNAs related to cancer stem cell properties (let-7 [29], miR-34a [30], and miR-141 [31]) and EMT (miR-200 family [32]). miR-34a is also implicated in microenvironmental remodeling for the inhibition of bone metastasis [33]. All of these targets and underlying mechanisms are thoroughly reviewed in [34]. Given the versatility of the nanodelivery platform described here, further studies that explore these miRNAs for therapy against metastasis are warranted.

## Supporting information

S1 File TargetScan predicted murine targets of mmu-miR-710. (XLSX)

S2 File MiRDB Predicted human targets of mmu-miR-710. (PDF)

S1 Fig List of miRNAs. List and level of expression of the 45 most highly differentially expressed miRNAs in the metastatic lesions relative to the primary tumors of mice bearing metastatic breast cancer isografts.
(TIF)

S2 Fig Functional annotation clustering of miR-710 predicted murine targets. The KEGG Pathways in Cancer annotation cluster is shown with the predicted targets marked by red stars.
(TIF)

S3 Fig Functional annotation clustering of miR-710 predicted human targets. The KEGG Pathways in Cancer annotation cluster is shown with the predicted targets marked by red stars.
(TIF)
