# Supplementary material for: MicroRNA-710 regulates multiple pathways of carcinogenesis in murine metastatic breast cancer
Source: PLoS One. 2019 Dec 13;14(12):e0226356. doi: 10.1371/journal.pone.0226356 (PMC6910689; doi:10.1371/journal.pone.0226356)
Supplement: S2 File — (PDF) [file pone.0226356.s002.pdf]

miRDB

There are 508 predicted targets for the submitted 22 nt long miRNA: ccaagucuuggggagaguugag

Return to Custom Prediction

Target Detail	Target Rank	Target Score	miRNA Name	Gene Symbol	Gene Description
Details	1	97	submission	CDK8	cyclin dependent kinase 8
Details	2	97	submission	HOXD13	homeobox D13
Details	3	96	submission	BEND4	BEN domain containing 4
Details	4	96	submission	HIPK3	homeodomain interacting protein kinase 3
Details	5	96	submission	DDX17	DEAD-box helicase 17
Details	6	95	submission	STT3B	STT3B, catalytic subunit of the oligosaccharyltransferase complex
Details	7	95	submission	ATXN7	ataxin 7
Details	8	95	submission	ZCCHC24	zinc finger CCHC-type containing 24
Details	9	95	submission	PPARGC1B	PPARG coactivator 1 beta
Details	10	95	submission	YTHDF1	YTH N6-methyladenosine RNA binding protein 1
Details	11	94	submission	LSM12	LSM12 homolog
Details	12	94	submission	ATP2C1	ATPase secretory pathway Ca2+ transporting 1
Details	13	94	submission	ARMCX2	armadillo repeat containing X-linked 2
Details	14	94	submission	GATM	glycine amidinotransferase
Details	15	94	submission	EPHA7	EPH receptor A7
Details	16	94	submission	C18orf25	chromosome 18 open reading frame 25
Details	17	93	submission	BNIP2	BCL2 interacting protein 2
Details	18	93	submission	CHSY1	chondroitin sulfate synthase 1
Details	19	93	submission	ASB3	ankyrin repeat and SOCS box containing 3
Details	20	93	submission	A2M	alpha-2-macroglobulin
Details	21	93	submission	KLF9	Kruppel like factor 9
Details	22	92	submission	FGF18	fibroblast growth factor 18
Details	23	92	submission	ZFP1	ZFP1 zinc finger protein
Details	24	92	submission	MEIOC	meiosis specific with coiled-coil domain
	25	92	submission		cytoplasmic polyadenylation element

Details				CPEB4	binding protein 4
Details	26	92	submission	RFX3	regulatory factor X3
Details	27	91	submission	ESR1	estrogen receptor 1
Details	28	91	submission	PGRMC2	progesterone receptor membrane component 2
Details	29	91	submission	SPIN1	spindlin 1
Details	30	91	submission	HSDL2	hydroxysteroid dehydrogenase like 2
Details	31	90	submission	CAPZA1	capping actin protein of muscle Z-line subunit alpha 1
Details	32	90	submission	TMEM248	transmembrane protein 248
Details	33	90	submission	ZFAND6	zinc finger AN1-type containing 6
Details	34	90	submission	LRRTM1	leucine rich repeat transmembrane neuronal 1
Details	35	90	submission	CDK13	cyclin dependent kinase 13
Details	36	89	submission	PRSS12	serine protease 12
Details	37	89	submission	MAGI1	membrane associated guanylate kinase, WW and PDZ domain containing 1
Details	38	89	submission	EIF2S1	eukaryotic translation initiation factor 2 subunit alpha
Details	39	89	submission	UBE2W	ubiquitin conjugating enzyme E2 W
Details	40	88	submission	ADAMTS3	ADAM metallopeptidase with thrombospondin type 1 motif 3
Details	41	88	submission	SKP2	S-phase kinase associated protein 2
Details	42	88	submission	TCF7L2	transcription factor 7 like 2
Details	43	88	submission	AZI2	5-azacytidine induced 2
Details	44	88	submission	EXOC6	exocyst complex component 6
Details	45	88	submission	GLB1	galactosidase beta 1
Details	46	88	submission	TNRC6B	trinucleotide repeat containing 6B
Details	47	88	submission	CDC14A	cell division cycle 14A
Details	48	88	submission	SOCS5	suppressor of cytokine signaling 5
Details	49	88	submission	HUWE1	HECT, UBA and WWE domain containing 1, E3 ubiquitin protein ligase
Details	50	87	submission	PPIP5K1	diphosphoinositol pentakisphosphate kinase 1
Details	51	87	submission	AIMP1	aminoacyl tRNA synthetase complex interacting multifunctional protein 1
Details	52	87	submission	PQLC3	PQ loop repeat containing 3
Details	53	87	submission	CHD6	chromodomain helicase DNA binding protein 6
Details	54	87	submission	SOX4	SRY-box 4
Details	55	86	submission	PSMC2	proteasome 26S subunit, ATPase 2

Details	56	86	submission	SCAI	suppressor of cancer cell invasion
Details	57	86	submission	ZNF385B	zinc finger protein 385B
Details	58	86	submission	CA13	carbonic anhydrase 13
Details	59	86	submission	GPM6B	glycoprotein M6B
Details	60	86	submission	ITGA6	integrin subunit alpha 6
Details	61	86	submission	PI4K2B	phosphatidylinositol 4-kinase type 2 beta
Details	62	86	submission	LYRM1	LYR motif containing 1
Details	63	86	submission	PWWP2A	PWWP domain containing 2A
Details	64	85	submission	WDFY3	WD repeat and FYVE domain containing 3
Details	65	85	submission	TLL1	tolloid like 1
Details	66	85	submission	MEX3C	mex-3 RNA binding family member C
Details	67	85	submission	MAP4K4	mitogen-activated protein kinase kinase kinase kinase 4
Details	68	85	submission	RLIM	ring finger protein, LIM domain interacting
Details	69	85	submission	DSG2	desmoglein 2
Details	70	85	submission	MPHOSPH9	M-phase phosphoprotein 9
Details	71	84	submission	SYNM	synemin
Details	72	84	submission	ITFG1	integrin alpha FG-GAP repeat containing 1
Details	73	84	submission	KDM5A	lysine demethylase 5A
Details	74	84	submission	MAP3K9	mitogen-activated protein kinase kinase kinase 9
Details	75	84	submission	PDGFRA	platelet derived growth factor receptor alpha
Details	76	84	submission	ITGB1	integrin subunit beta 1
Details	77	84	submission	BVES	blood vessel epicardial substance
Details	78	83	submission	SASH3	SAM and SH3 domain containing 3
Details	79	83	submission	TAL1	TAL bHLH transcription factor 1, erythroid differentiation factor
Details	80	83	submission	SYTL5	synaptotagmin like 5
Details	81	83	submission	COL11A1	collagen type XI alpha 1 chain
Details	82	83	submission	PRKG1	protein kinase cGMP-dependent 1
Details	83	83	submission	RELT	RELT, TNF receptor
Details	84	82	submission	FRK	fyn related Src family tyrosine kinase
Details	85	82	submission	LRAT	lecithin retinol acyltransferase
Details	86	82	submission	TXNRD1	thioredoxin reductase 1
Details	87	82	submission	PIP4P2	phosphatidylinositol-4,5-bisphosphate 4-phosphatase 2
Details	88	82	submission	NUAK2	NUAK family kinase 2
Details	89	82	submission	KCNG3	potassium voltage-gated channel modifier subfamily G member 3

Details	90	81	submission	ABCA1	ATP binding cassette subfamily A member 1
Details	91	81	submission	CCN2	cellular communication network factor 2
Details	92	81	submission	SKI	SKI proto-oncogene
Details	93	81	submission	NAPEPLD	N-acyl phosphatidylethanolamine phospholipase D
Details	94	81	submission	CENPC	centromere protein C
Details	95	81	submission	FAM199X	family with sequence similarity 199, X-linked
Details	96	81	submission	SENP6	SUMO specific peptidase 6
Details	97	80	submission	RSPRY1	ring finger and SPRY domain containing 1
Details	98	80	submission	SUB1	SUB1 homolog, transcriptional regulator
Details	99	80	submission	DAB2	DAB2, clathrin adaptor protein
Details	100	80	submission	INSIG1	insulin induced gene 1
Details	101	80	submission	IHH	Indian hedgehog signaling molecule
Details	102	79	submission	CCDC32	coiled-coil domain containing 32
Details	103	79	submission	GPCPD1	glycerophosphocholine phosphodiesterase 1
Details	104	79	submission	IL4R	interleukin 4 receptor
Details	105	79	submission	RIMKLB	ribosomal modification protein rimK like family member B
Details	106	79	submission	HSPA9	heat shock protein family A (Hsp70) member 9
Details	107	79	submission	GOLT1B	golgi transport 1B
Details	108	79	submission	CMIP	c-Maf inducing protein
Details	109	79	submission	PLCL1	phospholipase C like 1 (inactive)
Details	110	79	submission	PHF23	PHD finger protein 23
Details	111	79	submission	HOOK3	hook microtubule tethering protein 3
Details	112	79	submission	CCDC6	coiled-coil domain containing 6
Details	113	79	submission	CAST	calpastatin
Details	114	79	submission	LARP4B	La ribonucleoprotein domain family member 4B
Details	115	79	submission	ZNF543	zinc finger protein 543
Details	116	78	submission	FBXO11	F-box protein 11
Details	117	78	submission	GRIK5	glutamate ionotropic receptor kainate type subunit 5
Details	118	78	submission	LTN1	listerin E3 ubiquitin protein ligase 1
Details	119	78	submission	CNTN6	contactin 6
Details	120	78	submission	PLEKHA8	pleckstrin homology domain containing A8
Details	121	78	submission	GID4	GID complex subunit 4 homolog
Details	122	78	submission	AMOTL2	angiomotin like 2

Details	123	78	submission	MEF2C	myocyte enhancer factor 2C
Details	124	78	submission	XRN1	5'-3' exoribonuclease 1
Details	125	78	submission	MTHFD2L	methylenetetrahydrofolate dehydrogenase (NADP+ dependent) 2 like
Details	126	78	submission	SETD5	SET domain containing 5
Details	127	78	submission	TMEFF1	transmembrane protein with EGF like and two follistatin like domains 1
Details	128	78	submission	SOD2	superoxide dismutase 2
Details	129	78	submission	PDLIM5	PDZ and LIM domain 5
Details	130	78	submission	CHD5	chromodomain helicase DNA binding protein 5
Details	131	77	submission	KSR2	kinase suppressor of ras 2
Details	132	77	submission	TARDBP	TAR DNA binding protein
Details	133	77	submission	KCNJ2	potassium voltage-gated channel subfamily J member 2
Details	134	77	submission	USP48	ubiquitin specific peptidase 48
Details	135	77	submission	MASP1	mannan binding lectin serine peptidase 1
Details	136	77	submission	KCNE3	potassium voltage-gated channel subfamily E regulatory subunit 3
Details	137	77	submission	GRIK2	glutamate ionotropic receptor kainate type subunit 2
Details	138	77	submission	ITGA10	integrin subunit alpha 10
Details	139	77	submission	RASEF	RAS and EF-hand domain containing
Details	140	77	submission	MTO1	mitochondrial tRNA translation optimization 1
Details	141	77	submission	SRPRA	SRP receptor subunit alpha
Details	142	77	submission	TMEM170B	transmembrane protein 170B
Details	143	76	submission	DCP2	decapping mRNA 2
Details	144	76	submission	WNT2	Wnt family member 2
Details	145	76	submission	SGCB	sarcoglycan beta
Details	146	76	submission	FSBP	fibrinogen silencer binding protein
Details	147	76	submission	ZNF148	zinc finger protein 148
Details	148	76	submission	HPSE	heparanase
Details	149	76	submission	SYNC	syncoilin, intermediate filament protein
Details	150	76	submission	NRXN1	neurexin 1
Details	151	76	submission	ABR	ABR, RhoGEF and GTPase activating protein
Details	152	76	submission	RAD54B	RAD54 homolog B
Details	153	75	submission	ARL5B	ADP ribosylation factor like GTPase 5B
Details	154	75	submission	CD200	CD200 molecule
Details	155	75	submission	HECTD2	HECT domain E3 ubiquitin protein ligase 2

Details	156	75	submission	LRCH1	leucine rich repeats and calponin homology domain containing 1
Details	157	75	submission	LIN52	lin-52 DREAM MuvB core complex component
Details	158	75	submission	UBN2	ubinuclein 2
Details	159	75	submission	BACE2	beta-secretase 2
Details	160	75	submission	FRMD5	FERM domain containing 5
Details	161	75	submission	MED23	mediator complex subunit 23
Details	162	75	submission	PPP2R5A	protein phosphatase 2 regulatory subunit B'alpha
Details	163	75	submission	FAM155B	family with sequence similarity 155 member B
Details	164	74	submission	TMEM132D	transmembrane protein 132D
Details	165	74	submission	ZBED2	zinc finger BED-type containing 2
Details	166	74	submission	MICALCL	MICAL C-terminal like
Details	167	74	submission	PYROXD1	pyridine nucleotide-disulphide oxidoreductase domain 1
Details	168	74	submission	CHIC1	cysteine rich hydrophobic domain 1
Details	169	74	submission	ZNF772	zinc finger protein 772
Details	170	74	submission	FLRT3	fibronectin leucine rich transmembrane protein 3
Details	171	73	submission	MAPK6	mitogen-activated protein kinase 6
Details	172	73	submission	ANKH	ANKH inorganic pyrophosphate transport regulator
Details	173	73	submission	PARVG	parvin gamma
Details	174	73	submission	ALG2	ALG2, alpha-1,3/1,6-mannosyltransferase
Details	175	73	submission	C5orf49	chromosome 5 open reading frame 49
Details	176	73	submission	TBC1D12	TBC1 domain family member 12
Details	177	72	submission	DYNC1I1	dynein cytoplasmic 1 intermediate chain 1
Details	178	72	submission	ZFYVE26	zinc finger FYVE-type containing 26
Details	179	72	submission	SLC23A1	solute carrier family 23 member 1
Details	180	72	submission	RFX8	RFX family member 8, lacking RFX DNA binding domain
Details	181	72	submission	MMEL1	membrane metalloendopeptidase like 1
Details	182	72	submission	TBL1XR1	transducin beta like 1 X-linked receptor 1
Details	183	72	submission	UPP2	uridine phosphorylase 2
Details	184	72	submission	OSBPL11	oxysterol binding protein like 11
Details	185	72	submission	DPP8	dipeptidyl peptidase 8
Details	186	72	submission	JAG1	jagged 1
Details	187	72	submission	TRERF1	transcriptional regulating factor 1
Details	188	72	submission	THSD7B	thrombospondin type 1 domain containing 7B

Details	189	71	submission	KCMF1	potassium channel modulatory factor 1
Details	190	71	submission	PHF6	PHD finger protein 6
Details	191	71	submission	MS4A4A	membrane spanning 4-domains A4A
Details	192	71	submission	CDC5L	cell division cycle 5 like
Details	193	71	submission	OXR1	oxidation resistance 1
Details	194	71	submission	KPNA3	karyopherin subunit alpha 3
Details	195	71	submission	RALGDS	ral guanine nucleotide dissociation stimulator
Details	196	71	submission	ARG1	arginase 1
Details	197	71	submission	BCL6	BCL6, transcription repressor
Details	198	71	submission	EDEM3	ER degradation enhancing alpha-mannosidase like protein 3
Details	199	70	submission	EDAR	ectodysplasin A receptor
Details	200	70	submission	RDH10	retinol dehydrogenase 10
Details	201	70	submission	FGF19	fibroblast growth factor 19
Details	202	70	submission	AGO1	argonaute RISC catalytic component 1
Details	203	70	submission	KMT2D	lysine methyltransferase 2D
Details	204	70	submission	PLEKHG5	pleckstrin homology and RhoGEF domain containing G5
Details	205	70	submission	FAM78A	family with sequence similarity 78 member A
Details	206	70	submission	FIGN	fidgetin, microtubule severing factor
Details	207	70	submission	BDNF	brain derived neurotrophic factor
Details	208	70	submission	KDM6A	lysine demethylase 6A
Details	209	70	submission	ERP44	endoplasmic reticulum protein 44
Details	210	70	submission	OR13A1	olfactory receptor family 13 subfamily A member 1
Details	211	69	submission	RNASE7	ribonuclease A family member 7
Details	212	69	submission	BTG3	BTG anti-proliferation factor 3
Details	213	69	submission	MMP13	matrix metallopeptidase 13
Details	214	69	submission	ITPR3	inositol 1,4,5-trisphosphate receptor type 3
Details	215	69	submission	YIPF3	Yip1 domain family member 3
Details	216	69	submission	CDR1	cerebellar degeneration related protein 1
Details	217	69	submission	GPD2	glycerol-3-phosphate dehydrogenase 2
Details	218	69	submission	EBAG9	estrogen receptor binding site associated, antigen, 9
Details	219	69	submission	STIM2	stromal interaction molecule 2
Details	220	69	submission	CHST2	carbohydrate sulfotransferase 2
	221	69	submission	LRRFIP2	LRR binding FLII interacting protein 2

Details					
Details	222	68	submission	BAGE2	BAGE family member 2
Details	223	68	submission	BPTF	bromodomain PHD finger transcription factor
Details	224	68	submission	SOGA1	suppressor of glucose, autophagy associated 1
Details	225	68	submission	RANBP3	RAN binding protein 3
Details	226	68	submission	LPAR1	lysophosphatidic acid receptor 1
Details	227	68	submission	ST8SIA3	ST8 alpha-N-acetyl-neuraminide alpha-2,8-sialyltransferase 3
Details	228	68	submission	CXorf40A	chromosome X open reading frame 40A
Details	229	68	submission	MLLT3	MLLT3, super elongation complex subunit
Details	230	68	submission	SYT17	synaptotagmin 17
Details	231	68	submission	TRIM14	tripartite motif containing 14
Details	232	67	submission	PRTFDC1	phosphoribosyl transferase domain containing 1
Details	233	67	submission	ZNF652	zinc finger protein 652
Details	234	67	submission	CYB5B	cytochrome b5 type B
Details	235	67	submission	TTC37	tetratricopeptide repeat domain 37
Details	236	67	submission	CORO1C	coronin 1C
Details	237	67	submission	PHF14	PHD finger protein 14
Details	238	67	submission	KLHL14	kelch like family member 14
Details	239	67	submission	CELF2	CUGBP Elav-like family member 2
Details	240	67	submission	C17orf51	chromosome 17 open reading frame 51
Details	241	67	submission	ZNF706	zinc finger protein 706
Details	242	67	submission	PHACTR2	phosphatase and actin regulator 2
Details	243	66	submission	TRAK1	trafficking kinesin protein 1
Details	244	66	submission	GK2	glycerol kinase 2
Details	245	66	submission	MYSM1	Myb like, SWIRM and MPN domains 1
Details	246	66	submission	CXXC4	CXXC finger protein 4
Details	247	66	submission	LRRC17	leucine rich repeat containing 17
Details	248	66	submission	NDNF	neuron derived neurotrophic factor
Details	249	66	submission	ELAVL2	ELAV like RNA binding protein 2
Details	250	66	submission	PPP4R4	protein phosphatase 4 regulatory subunit 4
Details	251	66	submission	OLA1	Obg like ATPase 1
Details	252	66	submission	NEMP1	nuclear envelope integral membrane protein 1
Details	253	66	submission	ARHGAP21	Rho GTPase activating protein 21

Details	254	66	submission	PALLD	palladin, cytoskeletal associated protein
Details	255	66	submission	SNRPB2	small nuclear ribonucleoprotein polypeptide B2
Details	256	66	submission	TMEM184C	transmembrane protein 184C
Details	257	66	submission	MYORG	myogenesis regulating glycosidase (putative)
Details	258	65	submission	AAK1	AP2 associated kinase 1
Details	259	65	submission	EIF4E3	eukaryotic translation initiation factor 4E family member 3
Details	260	65	submission	STXBP5	syntaxin binding protein 5
Details	261	65	submission	CCNC	cyclin C
Details	262	65	submission	C10orf25	chromosome 10 open reading frame 25
Details	263	65	submission	CHD2	chromodomain helicase DNA binding protein 2
Details	264	65	submission	SLC5A8	solute carrier family 5 member 8
Details	265	65	submission	ITGBL1	integrin subunit beta like 1
Details	266	65	submission	GFPT1	glutamine--fructose-6-phosphate transaminase 1
Details	267	65	submission	ZNF37A	zinc finger protein 37A
Details	268	65	submission	VAPA	VAMP associated protein A
Details	269	65	submission	NAV2	neuron navigator 2
Details	270	65	submission	MREG	melanoregulin
Details	271	65	submission	RLF	rearranged L-myc fusion
Details	272	65	submission	MOCS2	molybdenum cofactor synthesis 2
Details	273	64	submission	DUSP11	dual specificity phosphatase 11
Details	274	64	submission	TTK	TTK protein kinase
Details	275	64	submission	DIO1	iodothyronine deiodinase 1
Details	276	64	submission	TEPSIN	TEPSIN, adaptor related protein complex 4 accessory protein
Details	277	64	submission	CRTC1	CREB regulated transcription coactivator 1
Details	278	64	submission	WDFY1	WD repeat and FYVE domain containing 1
Details	279	64	submission	TOR1AIP2	torsin 1A interacting protein 2
Details	280	64	submission	TM4SF18	transmembrane 4 L six family member 18
Details	281	64	submission	GON4L	gon-4 like
Details	282	63	submission	LRP6	LDL receptor related protein 6
Details	283	63	submission	GBP2	guanylate binding protein 2
Details	284	63	submission	TNIP3	TNFAIP3 interacting protein 3
Details	285	63	submission	HELZ	helicase with zinc finger
Details	286	63	submission	PELO	pelota mRNA surveillance and ribosome rescue factor
	287	63	submission	WDR92	WD repeat domain 92

Details					
Details	288	63	submission	C19orf12	chromosome 19 open reading frame 12
Details	289	63	submission	ZMYND8	zinc finger MYND-type containing 8
Details	290	63	submission	SLC25A17	solute carrier family 25 member 17
Details	291	63	submission	PPP6C	protein phosphatase 6 catalytic subunit
Details	292	63	submission	SOCS4	suppressor of cytokine signaling 4
Details	293	63	submission	ARMCX1	armadillo repeat containing X-linked 1
Details	294	62	submission	LEPROTL1	leptin receptor overlapping transcript like 1
Details	295	62	submission	ENDOD1	endonuclease domain containing 1
Details	296	62	submission	TEX261	testis expressed 261
Details	297	62	submission	PSENEN	presenilin enhancer, gamma-secretase subunit
Details	298	62	submission	INHBB	inhibin subunit beta B
Details	299	62	submission	CDKL1	cyclin dependent kinase like 1
Details	300	62	submission	CFL2	cofilin 2
Details	301	62	submission	SLC35E1	solute carrier family 35 member E1
Details	302	62	submission	CDKN2D	cyclin dependent kinase inhibitor 2D
Details	303	62	submission	AGAP1	ArfGAP with GTPase domain, ankyrin repeat and PH domain 1
Details	304	62	submission	E2F7	E2F transcription factor 7
Details	305	62	submission	PTK2	protein tyrosine kinase 2
Details	306	61	submission	SLC26A9	solute carrier family 26 member 9
Details	307	61	submission	RGPD1	RANBP2-like and GRIP domain containing 1
Details	308	61	submission	LHX8	LIM homeobox 8
Details	309	61	submission	ZNF208	zinc finger protein 208
Details	310	61	submission	CD5L	CD5 molecule like
Details	311	61	submission	ITPR2	inositol 1,4,5-trisphosphate receptor type 2
Details	312	61	submission	EFNA2	ephrin A2
Details	313	61	submission	DBNDD1	dysbindin domain containing 1
Details	314	61	submission	MID2	midline 2
Details	315	61	submission	NEO1	neogenin 1
Details	316	61	submission	CCDC141	coiled-coil domain containing 141
Details	317	61	submission	SLC7A5	solute carrier family 7 member 5
Details	318	61	submission	VLDLR	very low density lipoprotein receptor
Details	319	61	submission	TAB2	TGF-beta activated kinase 1 (MAP3K7) binding protein 2
	320	60	submission	CD1D	CD1d molecule

Details					
Details	321	60	submission	ZFP36L1	ZFP36 ring finger protein like 1
Details	322	60	submission	ERG	ETS transcription factor ERG
Details	323	60	submission	MPRIP	myosin phosphatase Rho interacting protein
Details	324	60	submission	NIPAL4	NIPA like domain containing 4
Details	325	60	submission	DBF4B	DBF4 zinc finger B
Details	326	60	submission	SLC10A1	solute carrier family 10 member 1
Details	327	60	submission	PTPN14	protein tyrosine phosphatase, non-receptor type 14
Details	328	60	submission	WNT10B	Wnt family member 10B
Details	329	60	submission	CELSR2	cadherin EGF LAG seven-pass G-type receptor 2
Details	330	60	submission	DPYSL2	dihydropyrimidinase like 2
Details	331	60	submission	MOXD1	monooxygenase DBH like 1
Details	332	60	submission	TDP2	tyrosyl-DNA phosphodiesterase 2
Details	333	60	submission	CIAO1	cytosolic iron-sulfur assembly component 1
Details	334	60	submission	DDX20	DEAD-box helicase 20
Details	335	60	submission	ACER2	alkaline ceramidase 2
Details	336	59	submission	PARD6B	par-6 family cell polarity regulator beta
Details	337	59	submission	SH3BGRL2	SH3 domain binding glutamate rich protein like 2
Details	338	59	submission	CNOT6	CCR4-NOT transcription complex subunit 6
Details	339	59	submission	GPR88	G protein-coupled receptor 88
Details	340	59	submission	SATB2	SATB homeobox 2
Details	341	59	submission	UNC45B	unc-45 myosin chaperone B
Details	342	59	submission	TNKS2	tankyrase 2
Details	343	59	submission	TFRC	transferrin receptor
Details	344	59	submission	LYSMD4	LysM domain containing 4
Details	345	58	submission	SHROOM2	shroom family member 2
Details	346	58	submission	ZNF75A	zinc finger protein 75a
Details	347	58	submission	PPP1R17	protein phosphatase 1 regulatory subunit 17
Details	348	58	submission	CTC1	CST telomere replication complex component 1
Details	349	58	submission	MYOZ2	myozenin 2
Details	350	58	submission	TNNI3K	TNNI3 interacting kinase
Details	351	58	submission	FPGT-TNNI3K	FPGT-TNNI3K readthrough
Details	352	58	submission	C6orf226	chromosome 6 open reading frame 226

Details	353	58	submission	KIRREL1	kirre like nephrin family adhesion molecule 1
Details	354	58	submission	PARD3	par-3 family cell polarity regulator
Details	355	58	submission	ZMYM1	zinc finger MYM-type containing 1
Details	356	58	submission	ZC3H14	zinc finger CCCH-type containing 14
Details	357	58	submission	SP4	Sp4 transcription factor
Details	358	58	submission	CD28	CD28 molecule
Details	359	58	submission	RNMT	RNA guanine-7 methyltransferase
Details	360	58	submission	PNMA8B	PNMA family member 8B
Details	361	58	submission	OXA1L	OXA1L, mitochondrial inner membrane protein
Details	362	58	submission	SUN1	Sad1 and UNC84 domain containing 1
Details	363	58	submission	MED22	mediator complex subunit 22
Details	364	58	submission	MTR	5-methyltetrahydrofolate-homocysteine methyltransferase
Details	365	58	submission	SMO	smoothened, frizzled class receptor
Details	366	57	submission	CTNNBIP1	catenin beta interacting protein 1
Details	367	57	submission	NACC2	NACC family member 2
Details	368	57	submission	COLEC12	collectin subfamily member 12
Details	369	57	submission	SRPK1	SRSF protein kinase 1
Details	370	57	submission	TRPM1	transient receptor potential cation channel subfamily M member 1
Details	371	57	submission	ERCC6L2	ERCC excision repair 6 like 2
Details	372	57	submission	C17orf58	chromosome 17 open reading frame 58
Details	373	57	submission	UBASH3B	ubiquitin associated and SH3 domain containing B
Details	374	57	submission	DIP2B	disco interacting protein 2 homolog B
Details	375	57	submission	TOMM20	translocase of outer mitochondrial membrane 20
Details	376	57	submission	MYPOP	Myb related transcription factor, partner of profilin
Details	377	57	submission	IRF2BP2	interferon regulatory factor 2 binding protein 2
Details	378	57	submission	ADAMTS5	ADAM metallopeptidase with thrombospondin type 1 motif 5
Details	379	57	submission	RASSF6	Ras association domain family member 6
Details	380	57	submission	SLC30A4	solute carrier family 30 member 4
Details	381	57	submission	SUSD6	sushi domain containing 6
Details	382	57	submission	C5orf66	chromosome 5 open reading frame 66
Details	383	57	submission	ZNF611	zinc finger protein 611
Details	384	56	submission	RGPD2	RANBP2-like and GRIP domain containing 2

Details	385	56	submission	UGCG	UDP-glucose ceramide glucosyltransferase
Details	386	56	submission	TCAIM	T cell activation inhibitor, mitochondrial
Details	387	56	submission	ASXL1	ASXL transcriptional regulator 1
Details	388	56	submission	MYCL	MYCL proto-oncogene, bHLH transcription factor
Details	389	56	submission	BRWD1	bromodomain and WD repeat domain containing 1
Details	390	56	submission	ARHGEF38	Rho guanine nucleotide exchange factor 38
Details	391	56	submission	ASAP3	ArfGAP with SH3 domain, ankyrin repeat and PH domain 3
Details	392	56	submission	LIX1	limb and CNS expressed 1
Details	393	56	submission	FHOD3	formin homology 2 domain containing 3
Details	394	56	submission	FOXO1	forkhead box O1
Details	395	56	submission	GPC6	glypican 6
Details	396	56	submission	FCRL2	Fc receptor like 2
Details	397	56	submission	RPL7L1	ribosomal protein L7 like 1
Details	398	56	submission	MEX3B	mex-3 RNA binding family member B
Details	399	55	submission	STX2	syntaxin 2
Details	400	55	submission	FAM204A	family with sequence similarity 204 member A
Details	401	55	submission	CUL3	cullin 3
Details	402	55	submission	TRIM26	tripartite motif containing 26
Details	403	55	submission	IGSF3	immunoglobulin superfamily member 3
Details	404	55	submission	PIGM	phosphatidylinositol glycan anchor biosynthesis class M
Details	405	55	submission	SCARB2	scavenger receptor class B member 2
Details	406	55	submission	HEATR1	HEAT repeat containing 1
Details	407	55	submission	DNAJC24	DnaJ heat shock protein family (Hsp40) member C24
Details	408	55	submission	OPCML	opioid binding protein/cell adhesion molecule like
Details	409	55	submission	PLEKHG4	pleckstrin homology and RhoGEF domain containing G4
Details	410	55	submission	RPIA	ribose 5-phosphate isomerase A
Details	411	55	submission	GABRA4	gamma-aminobutyric acid type A receptor alpha4 subunit
Details	412	55	submission	PLEKHA1	pleckstrin homology domain containing A1
Details	413	55	submission	CNTD2	cyclin N-terminal domain containing 2
Details	414	55	submission	KCNA1	potassium voltage-gated channel subfamily A member 1
Details	415	55	submission	ACTR3	ARP3 actin related protein 3 homolog

Details	416	55	submission	AGFG1	ArfGAP with FG repeats 1
Details	417	54	submission	AQP11	aquaporin 11
Details	418	54	submission	CYLC2	cylicin 2
Details	419	54	submission	FGGY	FGGY carbohydrate kinase domain containing
Details	420	54	submission	RAB14	RAB14, member RAS oncogene family
Details	421	54	submission	KPNA1	karyopherin subunit alpha 1
Details	422	54	submission	HDDC3	HD domain containing 3
Details	423	54	submission	FAM49A	family with sequence similarity 49 member A
Details	424	54	submission	IMMP1L	inner mitochondrial membrane peptidase subunit 1
Details	425	54	submission	KAT7	lysine acetyltransferase 7
Details	426	54	submission	FSHB	follicle stimulating hormone subunit beta
Details	427	54	submission	DCLK1	doublecortin like kinase 1
Details	428	54	submission	LYRM2	LYR motif containing 2
Details	429	54	submission	LSAMP	limbic system associated membrane protein
Details	430	54	submission	PRSS35	serine protease 35
Details	431	54	submission	PIK3C2A	phosphatidylinositol-4-phosphate 3-kinase catalytic subunit type 2 alpha
Details	432	54	submission	ABCD3	ATP binding cassette subfamily D member 3
Details	433	53	submission	UBE2H	ubiquitin conjugating enzyme E2 H
Details	434	53	submission	IL1RAP	interleukin 1 receptor accessory protein
Details	435	53	submission	RELB	RELB proto-oncogene, NF-kB subunit
Details	436	53	submission	BMPR2	bone morphogenetic protein receptor type 2
Details	437	53	submission	ZNF582	zinc finger protein 582
Details	438	53	submission	ENOX2	ecto-NOX disulfide-thiol exchanger 2
Details	439	53	submission	ITGA4	integrin subunit alpha 4
Details	440	53	submission	CAB39	calcium binding protein 39
Details	441	53	submission	CACNA1G	calcium voltage-gated channel subunit alpha1 G
Details	442	53	submission	RC3H1	ring finger and CCCH-type domains 1
Details	443	53	submission	ST6GALNAC4	ST6 N-acetylgalactosaminide alpha-2,6-sialyltransferase 4
Details	444	53	submission	MARCH7	membrane associated ring-CH-type finger 7
Details	445	53	submission	XPO7	exportin 7
Details	446	53	submission	ZRANB1	zinc finger RANBP2-type containing 1
Details	447	53	submission	DRAM1	DNA damage regulated autophagy modulator 1

Details	448	53	submission	FHIT	fragile histidine triad
Details	449	53	submission	GGA3	golgi associated, gamma adaptin ear containing, ARF binding protein 3
Details	450	53	submission	DEDD2	death effector domain containing 2
Details	451	53	submission	PHC3	polyhomeotic homolog 3
Details	452	52	submission	GSTM4	glutathione S-transferase mu 4
Details	453	52	submission	NRAS	NRAS proto-oncogene, GTPase
Details	454	52	submission	FKBP1B	FKBP prolyl isomerase 1B
Details	455	52	submission	SRSF2	serine and arginine rich splicing factor 2
Details	456	52	submission	ABCC3	ATP binding cassette subfamily C member 3
Details	457	52	submission	AP1S2	adaptor related protein complex 1 subunit sigma 2
Details	458	52	submission	TMEM168	transmembrane protein 168
Details	459	52	submission	PTPRO	protein tyrosine phosphatase, receptor type O
Details	460	52	submission	CRIM1	cysteine rich transmembrane BMP regulator 1
Details	461	52	submission	GTF2A1	general transcription factor IIA subunit 1
Details	462	52	submission	BRD3	bromodomain containing 3
Details	463	52	submission	PKHD1	PKHD1, fibrocystin/polyductin
Details	464	52	submission	RNF182	ring finger protein 182
Details	465	52	submission	ROBO3	roundabout guidance receptor 3
Details	466	52	submission	PRKCSH	protein kinase C substrate 80K-H
Details	467	52	submission	FRS2	fibroblast growth factor receptor substrate 2
Details	468	52	submission	SLC16A7	solute carrier family 16 member 7
Details	469	52	submission	ACP1	acid phosphatase 1
Details	470	52	submission	CFAP43	cilia and flagella associated protein 43
Details	471	52	submission	IPCEF1	interaction protein for cytohesin exchange factors 1
Details	472	51	submission	THUMPD1	THUMP domain containing 1
Details	473	51	submission	STX17	syntaxin 17
Details	474	51	submission	MSANTD3-TMEFF1	MSANTD3-TMEFF1 readthrough
Details	475	51	submission	HCFC2	host cell factor C2
Details	476	51	submission	HEXIM1	HEXIM P-TEFb complex subunit 1
Details	477	51	submission	CACTIN	cactin, spliceosome C complex subunit
Details	478	51	submission	PPM1E	protein phosphatase, Mg2+/Mn2+ dependent 1E
Details	479	51	submission	DCTN4	dynactin subunit 4

Details	480	51	submission	CBLL1	Cbl proto-oncogene like 1
Details	481	51	submission	TIMP2	TIMP metallopeptidase inhibitor 2
Details	482	51	submission	YBX1	Y-box binding protein 1
Details	483	51	submission	LCOR	ligand dependent nuclear receptor corepressor
Details	484	51	submission	CLUAP1	clusterin associated protein 1
Details	485	51	submission	PIK3R5	phosphoinositide-3-kinase regulatory subunit 5
Details	486	51	submission	NUP160	nucleoporin 160
Details	487	51	submission	ITSN1	intersectin 1
Details	488	51	submission	RAB18	RAB18, member RAS oncogene family
Details	489	51	submission	SRCIN1	SRC kinase signaling inhibitor 1
Details	490	51	submission	NFIB	nuclear factor I B
Details	491	51	submission	PHLDA1	pleckstrin homology like domain family A member 1
Details	492	51	submission	ADAM17	ADAM metallopeptidase domain 17
Details	493	51	submission	SERBP1	SERPINE1 mRNA binding protein 1
Details	494	51	submission	SYT14	synaptotagmin 14
Details	495	51	submission	ZNF627	zinc finger protein 627
Details	496	50	submission	BCL11A	BCL11A, BAF complex component
Details	497	50	submission	SLITRK4	SLIT and NTRK like family member 4
Details	498	50	submission	C5orf24	chromosome 5 open reading frame 24
Details	499	50	submission	UNC5D	unc-5 netrin receptor D
Details	500	50	submission	SGIP1	SH3 domain GRB2 like endophilin interacting protein 1
Details	501	50	submission	HES2	hes family bHLH transcription factor 2
Details	502	50	submission	TYW1	tRNA-yW synthesizing protein 1 homolog
Details	503	50	submission	A4GNT	alpha-1,4-N-acetylglucosaminyltransferase
Details	504	50	submission	DIS3	DIS3 homolog, exosome endoribonuclease and 3'-5' exoribonuclease
Details	505	50	submission	KLHL20	kelch like family member 20
Details	506	50	submission	LHX1	LIM homeobox 1
Details	507	50	submission	RPRD1A	regulation of nuclear pre-mRNA domain containing 1A
Details	508	50	submission	TYW1B	tRNA-yW synthesizing protein 1 homolog B
