# Supplementary figures and images for: MicroRNA-710 regulates multiple pathways of carcinogenesis in murine metastatic breast cancer
Source: PLoS One. 2019 Dec 13;14(12):e0226356. doi: 10.1371/journal.pone.0226356 (PMC6910689; doi:10.1371/journal.pone.0226356)

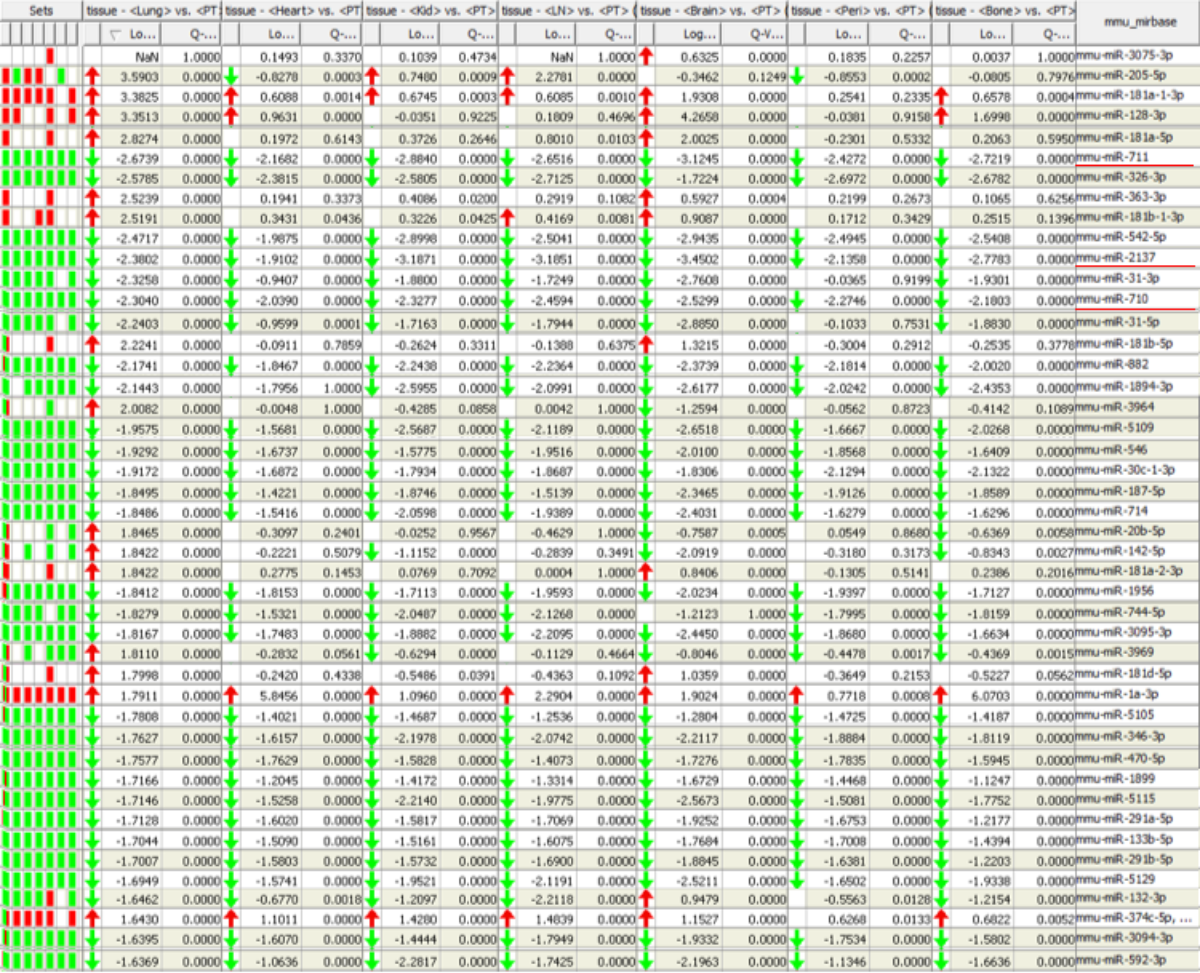

Supplement: S1 Fig — List and level of expression of the 45 most highly differentially expressed miRNAs in the metastatic lesions relative to the primary tumors of mice bearing metastatic breast cancer isografts. (TIF) [file pone.0226356.s003.tif]

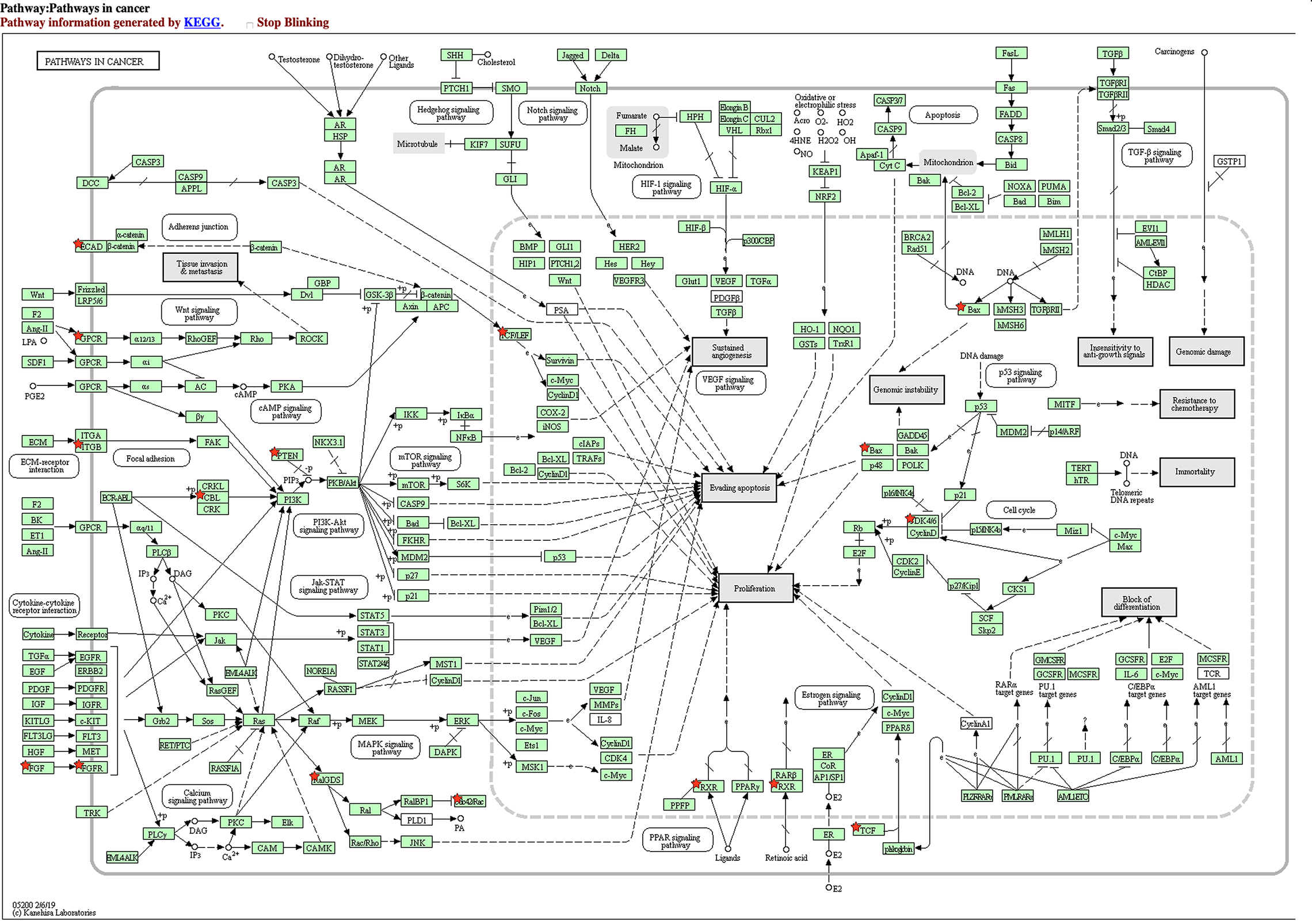

Supplement: S2 Fig — The KEGG Pathways in Cancer annotation cluster is shown with the predicted targets marked by red stars. (TIF) [file pone.0226356.s004.tif]

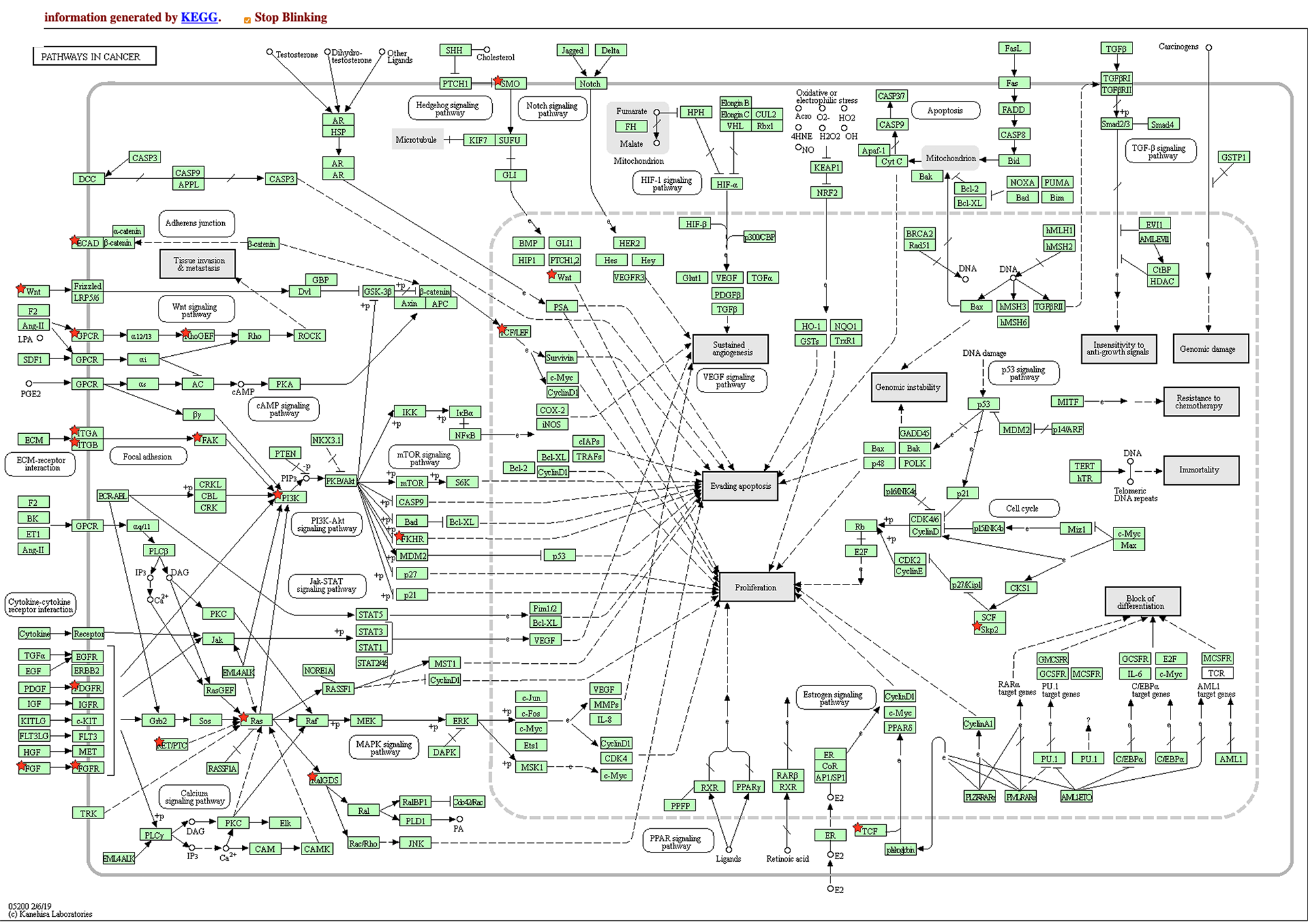

Supplement: S3 Fig — The KEGG Pathways in Cancer annotation cluster is shown with the predicted targets marked by red stars. (TIF) [file pone.0226356.s005.tif]
